# Hyperspectral Classification for Chinese Medicinal Herbs via Multi-Perspective Feature Extraction and Information-Theoretic Fusion

**DOI:** 10.3390/e28090973

**Published:** 2026-09-02

**Authors:** Zifei Li, Wei Huang, Ning Xu

**Affiliations:** 1 CUST-ITMO Joint Institute of Optics and Fine Mechanics, Changchun University of Science and Technology, Changchun 130022, China; 2 College of Computer Science and Technology, Zhejiang University of Technology, Hangzhou 310023, China; 3 College of Pharmaceutical Science, Zhejiang University of Technology, Huzhou 313200, China

**Keywords:** Chinese medicinal herbs, hyperspectral classification, multiview learning

## Abstract

Accurate and efficient classification of the origin of Chinese medicinal herbs is crucial for ensuring their quality, safety, and efficacy. This study takes the Chinese medicinal herb *Angelica dahurica* as an example for research. Hyperspectral reflectance data, which provides reflectance values of samples at different wavelengths, offers an effective way to characterize Chinese medicinal herbs. Hyperspectral reflectance data can be viewed as tabular data, allowing feature extraction via tabular data feature extraction methods; it can also be viewed as a reflectance sequence, enabling feature extraction by borrowing time-series feature extraction methods. That is, multiview learning can be applied to classify hyperspectral data. In this paper, an autoencoder (AE), a 1D convolutional neural network (1DCNN), and a Gated Recurrent Unit (GRU) are used for feature extraction. Then, a fusion network consisting of a linear transformation, a bilinear transformation, and a compression unit is proposed for feature fusion, and the feature fusion is based on the Information Bottleneck criterion. The fused features are fed into a fully connected neural network (the classifier) to perform the classification of the Chinese medicinal herb. Experimental results on *Angelica dahurica* data show that integrating features from different views effectively improves classification accuracy. Multiview classification can serve as a method for the classification of Chinese medicinal herbs.

## 1. Introduction

Traditional Chinese Medicine constitutes a vital component of the global healthcare system, and its clinical efficacy fundamentally depends on the quality of the Chinese medicinal herbs (CMHs) used. The quality of CMHs is influenced by multiple factors, including the harvesting season, processing techniques, and, most significantly, the geographical environment. Variations in climate, soil composition, and water conditions across different regions may lead to distinct chemical and pharmacological profiles of the same medicinal herb species [1]. Consequently, geographical origin is a critical determinant of CMH quality and therapeutic consistency. The ongoing development of the CMH industry creates an urgent need for accurate, rapid, and non-destructive methods for origin classification, which is paramount for enhancing quality control, ensuring clinical efficacy, and promoting the standardization of Traditional Chinese Medicine.

Traditional methodologies for CMH origin classification primarily encompass morphological identification and chemical analysis [2,3]. Morphological techniques, while foundational, are highly subjective, relying heavily on the expertise of trained practitioners, and often fail to discriminate between samples with subtle inter-regional variations. Chemical methods provide precise compositional information. However, they usually require complex sample pretreatment, involve expensive instrumentation, and are inherently destructive, making them impractical for large-scale and/or rapid classification requirements. These limitations significantly hinder the integration of such methods into modern, high-throughput Traditional Chinese Medicine quality control systems.

Hyperspectral imaging technology introduces a non-destructive paradigm for CMH origin classification. Hyperspectral reflectance data captures continuous spectral information on CMHs, spanning hundreds of narrow, contiguous wavelength bands. Hyperspectral reflectance data corresponds to a complete spectral signature, which encodes rich information about the herb’s physical structure and chemical composition [4]. For CMHs that are visually similar but originate from different locations, hyperspectral data can detect subtle internal differences imperceptible to the human eye, thereby enabling accurate discrimination. These features have established hyperspectral imaging as a prominent research tool in the field of CMH authentication and origin classification.

Several studies have validated the applicability of hyperspectral imaging for CMH origin classification. For example, hyperspectral technology alongside traditional machine learning classifiers has been applied for origin classification of the medicinal herbs *Atractylodes macrocephala* [5], *Astragalus membranaceus* [6], and *Chrysanthemum morifolium* [7], demonstrating the effectiveness and general applicability of hyperspectral technology for CMH origin classification.

Nevertheless, the effective utilization of hyperspectral data is challenged by its inherent characteristics: high dimensionality, strong inter-band correlation, and significant data redundancy. Traditional machine learning approaches applied to hyperspectral data classification, such as support vector machines (SVMs) [8] and Random Forests (RFs) [9], often depend on manual feature engineering. This process may not fully exploit the rich information hidden in the data, potentially leading to inferior classification accuracy. Deep learning methods provide a powerful tool to address these challenges. Models like convolutional neural networks (CNNs) and recurrent neural networks (RNNs) have been successfully applied to hyperspectral data analysis, enabling automatic feature extraction and demonstrating promising performance in various classification tasks [10,11].

Several deep-learning-based approaches for hyperspectral-based CMH classification have been proposed in recent years. For example, logistic regression, support vector machine, convolutional neural network, and recurrent neural network models for the identification of *Radix glycyrrhizae* using hyperspectral data were compared [12]. Hyperspectral imaging integrated with convolutional neural networks to distinguish 12 varieties of *Fritillaria thunbergii* was reported in [13]. A fully connected convolutional neural network based on hyperspectral images for the identification of *P. ginseng* growth years was studied in [14]. Terahertz spectroscopy combined with a CNN has been utilized for origin discrimination of *Panax notoginseng* [15]. 

These studies verify the superiority of deep-learning-based methods, but most of these studies extract features from a single perspective. Single-view feature extraction may lead to incomplete information utilization, limited model robustness, and lower classification accuracy for complex samples.

Hyperspectral reflectance data can be viewed as tabular data, allowing feature extraction via tabular data feature extraction methods; it can also be viewed as a reflectance sequence, enabling feature extraction by borrowing time-series feature extraction methods. That is, multiview learning can be applied to classify hyperspectral data. In this paper, an autoencoder (AE), a 1D convolutional neural network (1DCNN), and a Gated Recurrent Unit (GRU) are used for feature extraction. Then, a fusion network consisting of a linear transformation, a bilinear transformation, and a compression unit is proposed for feature fusion. The linear transformation accounts for linear combination of features. The bilinear transformation considers correlation among features, and it can model local pairwise feature interactions. The compression unit integrates all the above relationships and compresses the feature dimension. The feature fusion is based on the Information Bottleneck (IB) criterion [16,17,18], which compresses redundant information while preserving as much information as possible about the classification task. The fused features are fed into a fully connected neural network (the classifier) to perform the classification of a Chinese medicinal herb. Experimental results on *Angelica dahurica* data show that integrating features from different views effectively improves classification accuracy.

## 2. Data Description

### 2.1. Data Acquisition

This study adopted *Angelica dahurica* data collected by the research team of Prof. Ning Xu (College of Pharmaceutical Science, Zhejiang University of Technology, Huzhou 313200, Zhejiang, China). *Angelica dahurica* originating from five different provinces of China, namely Anhui, Zhejiang, Sichuan, Hebei, and Henan, were collected, with 1361, 1278, 969, 1012, and 1046 samples respectively (labeled 1–5 by origin). The collected *Angelica dahurica* were sliced, and all samples were approximately 3 mm thick.

For hyperspectral data acquisition, a system comprising an ImSpector N17E spectrograph (Spectral Imaging Ltd., Oulu, Finland), a 326 × 256-pixel Xeva 992 camera (Xenics, Leuven, Belgium), an OLES22 lens (Specim, Oulu, Finland), two 150 W tungsten halogen lamps (Illumination Technologies Inc., New York, NY, USA), and a stepper-motor-driven conveyor belt (Isuzu Optics Corp., Taiwan, China) was used, with acquisition controlled by Spectral-Image-Xenics 17E software (Isuzu Optics Corp., Taiwan, China) and parameters set to conveyor speed 9 mm/s, exposure time 3 ms, and camera lens height 10 cm. The finally collected hyperspectral data was the hyperspectral reflectance data of each sample. It was dimensionally reduced in the spatial domain and only contained 1D spectral information. The final reflectance data for each sample had 256 dimensions and covered a wavelength range of 874.41 nm to 1733.91 nm.

The whole dataset was randomly divided into a training set and a testing set with proportions of 80% and 20% respectively.

### 2.2. Data Visualization and Preprocessing

#### 2.2.1. t-SNE Data Visualization

t-SNE (t-Distributed Stochastic Neighbor Embedding) is a powerful probability-based nonlinear dimensionality reduction algorithm, serving as a critical tool for visualizing high-dimensional data. Its core objective is to map high-dimensional data (e.g., the hyperspectral reflectance data in this study) to a low-dimensional space (typically 2D or 3D) while maximally preserving the local neighborhood structure of the high-dimensional data points, thereby enabling intuitive visualization of clustering characteristics of samples from distinct categories.

The fundamental principle of t-SNE is briefly summarized as follows: First, the conditional probability $p_{j|i}$ that any two points $x_{i}$ and $x_{j}$ in the high-dimensional space are mutual neighbors is calculated as

$$p_{j|i}=\frac{\exp(-{\left\|x_{i}-x_{j}\right\|}^{2}/2\sigma_{i}^{2})}{\sum_{k\neq i}\exp(-{\left\|x_{i}-x_{k}\right\|}^{2}/2\sigma_{i}^{2})},$$

where $\sigma_{i}$ denotes the local bandwidth parameter of point $x_{i}$, adaptively controlling the neighborhood range. Next, global similarity between pairs of points is quantified via joint probability $p_{ij}=(p_{j|i}+p_{i|j})/2n$ (*n* = total samples), an analogous $q_{ij}$ is defined in a low dimension, and the KL divergence between $p_{ij}$ and $q_{ij}$ is minimized for low-dimensional embedding.

**Figure 1:**
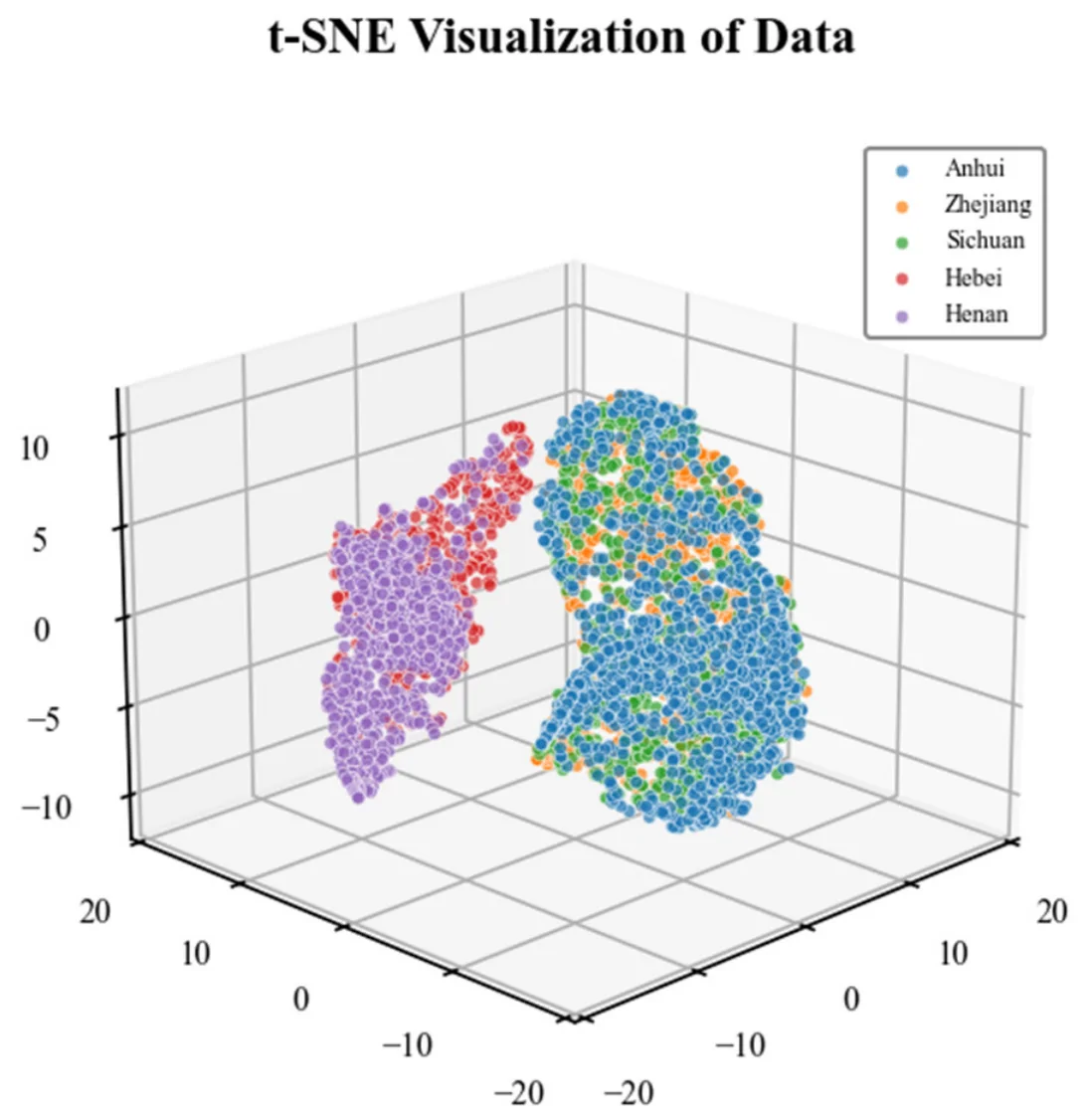
3D t-SNE visualization of hyperspectral data from *Angelica dahurica*.

In this study, t-SNE reduces 256-dimensional hyperspectral reflectance data (each sample has 256 wavelength-corresponding features) to 3 dimensions, yielding 3 non-physical features per sample corresponding to the X, Y, and Z axes of a 3D coordinate system. In Figure 1, samples are visualized using these dimensions, colored by origin (Anhui, Zhejiang, Sichuan, Hebei, Henan) for differentiation. The 3D scatter plot shows heavy overlap among samples from different regions, especially adjacent provinces, which indicates that the hyperspectral data itself has insufficient inter-class differences, and the classification task is challenging. Thus, feature extraction is crucial for the classification task.

#### 2.2.2. Data Preprocessing

**Figure 2:**
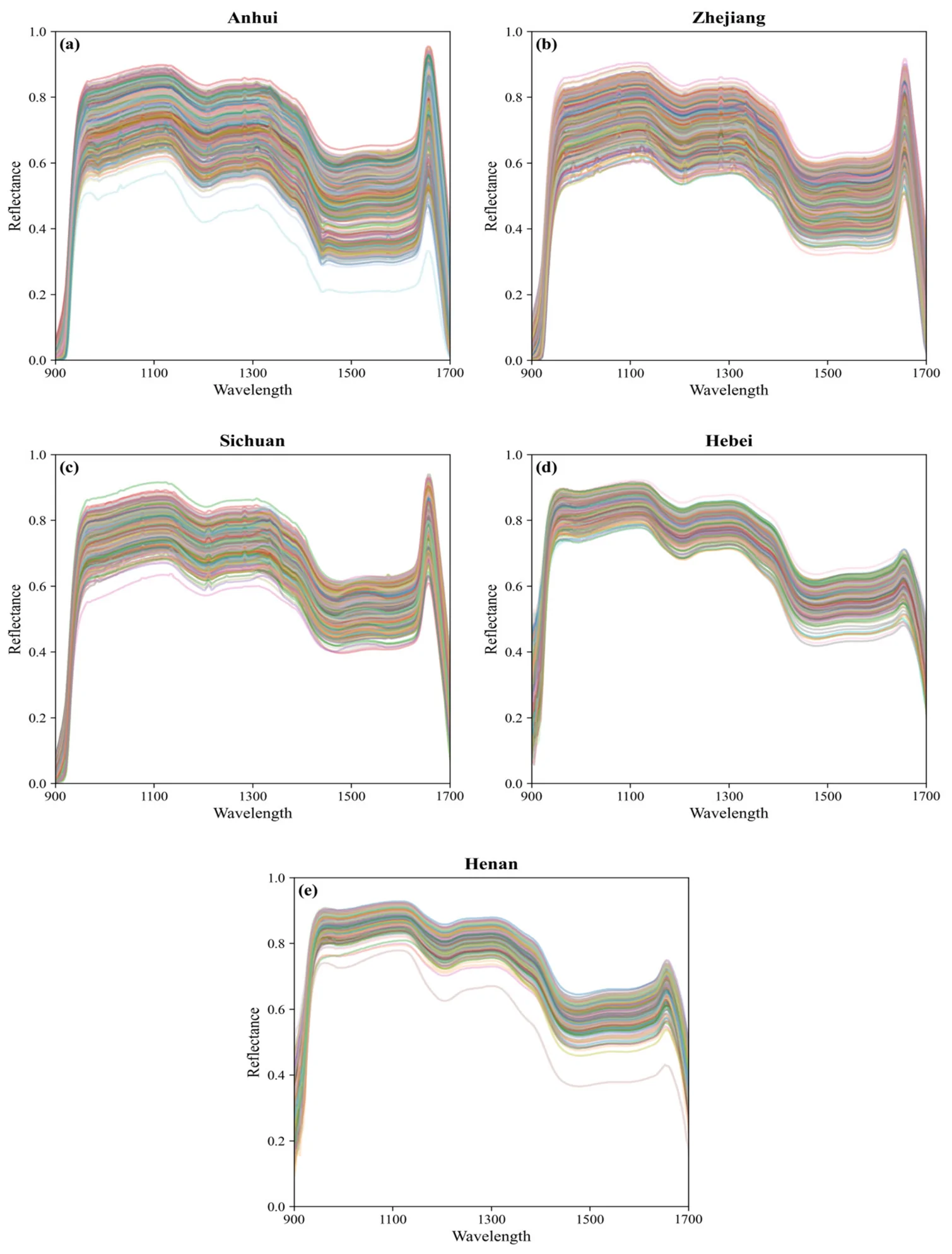
Hyperspectral curves of samples from different producing areas: (**a**) Anhui, (**b**) Zhejiang, (**c**) Sichuan, (**d**) Hebei, and (**e**) Henan.

The photodetectors of hyperspectral sensors exhibit different sensitivity across different wavelength bands. Typically, the signal strength is weaker at both ends of the spectrum, resulting in lower reflectance and higher noise levels, which may be removed during use. The reflectance data for the samples is shown in Figure 2, As we can see from the figure, this dataset also exhibits the abovementioned characteristics. Therefore, we perform preprocessing of the data by removing 20 dimensions from the front and 20 dimensions from the end; the remaining data dimension is 216 for each sample.

## 3. Method

### 3.1. Framework of the Whole Method

**Figure 3:**
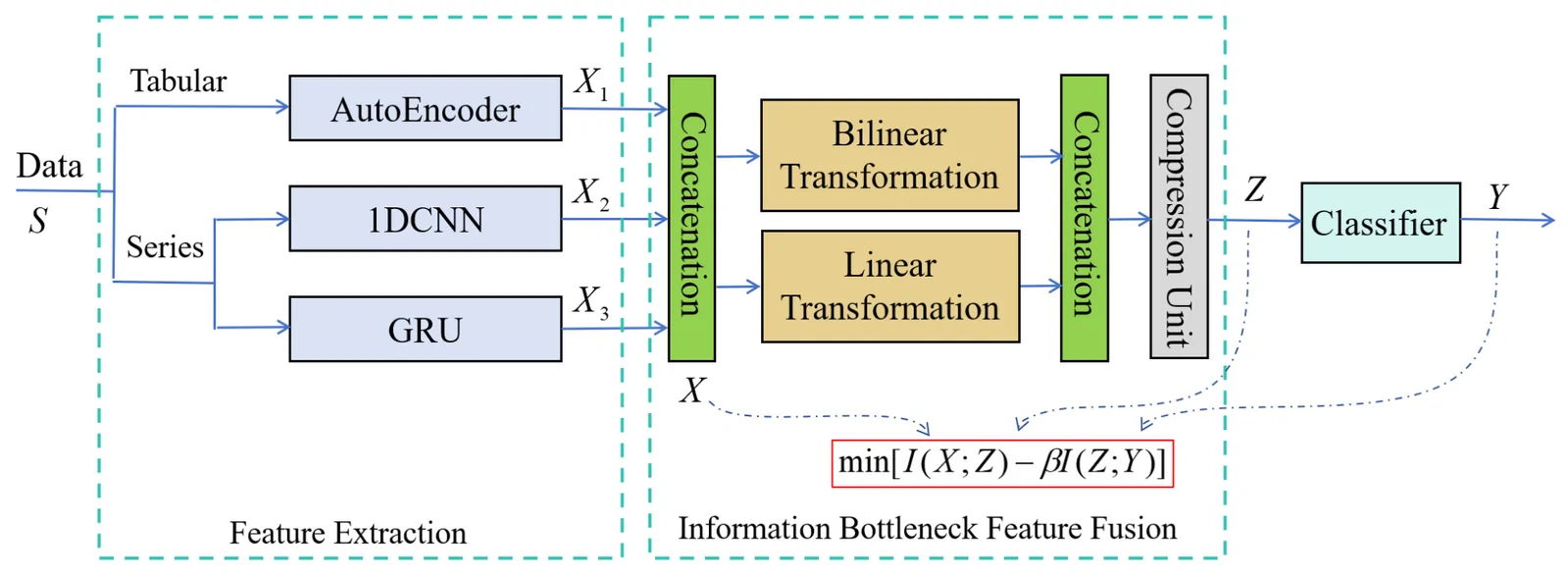
The framework of the whole method, which consists of three modules: a feature extraction module, an information bottleneck feature fusion module, and a classifier module.

The overall framework of the proposed method is shown in Figure 3, which consists of a feature extraction module, a feature fusion module, and a classifier. Each component is described in detail as follows.

### 3.2. Feature Extraction Module

After the preprocessing described in the previous section, each hyperspectral reflectance data sample has 216 dimensions. Hyperspectral reflectance data can be viewed as tabular data, allowing feature extraction via tabular data feature extraction methods; it can also be viewed as a reflectance sequence, enabling feature extraction by borrowing time-series feature extraction methods.

In this paper, we use an autoencoder (commonly used for tabular data feature extraction), as well as a 1DCNN and GRU (commonly used for time-series feature extraction), to extract features. Among them, the 1DCNN slides a convolution kernel along the wavelength dimension, mainly extracting short-term patterns and local trends in local spectral segments, while the GRU selectively remembers and forgets information through a gating mechanism, enabling it to capture long-distance dependencies. These two have good complementarity. The autoencoder maps the entire data vector to a fixed-length feature vector through an encoder, capturing the overall statistical characteristics of the data and achieving low-dimensional compression of the data, which also has certain complementarity with the above two feature extractors. These three methods for feature extraction can comprehensively extract data features from different perspectives. Employing multiple different feature extraction methods and then performing feature fusion is a typical multi-view learning paradigm.

Since these three feature extraction methods are widely applied neural network models, their specific principles are not described here. The specific network structures used in this paper are given below.

**Figure 4:**
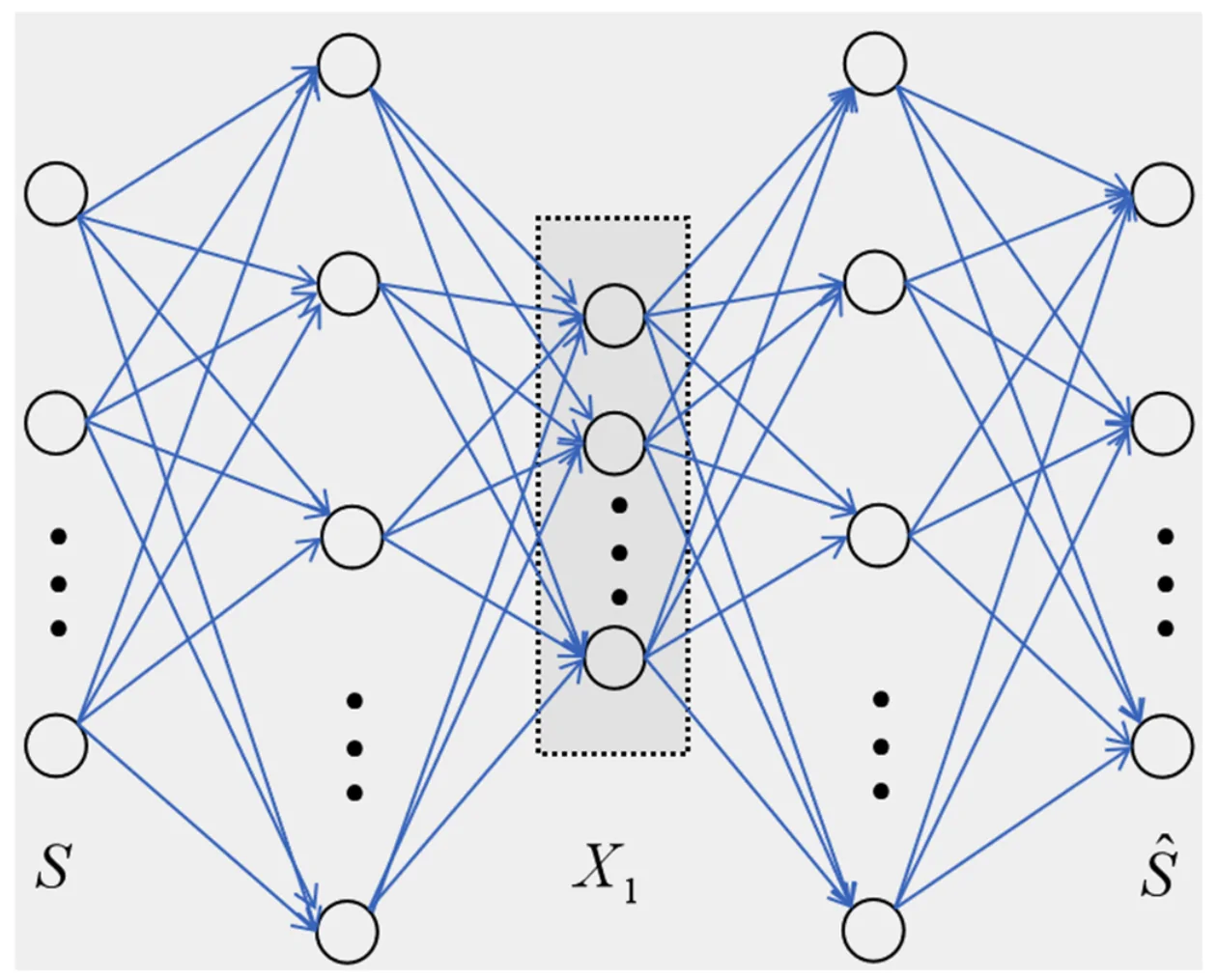
Architecture of autoencoder.

Both the encoder and decoder of the autoencoder (AE) are implemented as Multi-Layer Perceptrons (MLPs) with a single hidden layer, as shown in Figure 4. Specifically, the autoencoder takes 216-dimensional data as its input and outputs 216-dimensional reconstructed data, with the hidden-layer dimension set to 108. The dimension of the compressed feature vector extracted by the autoencoder is 20.

**Figure 5:**
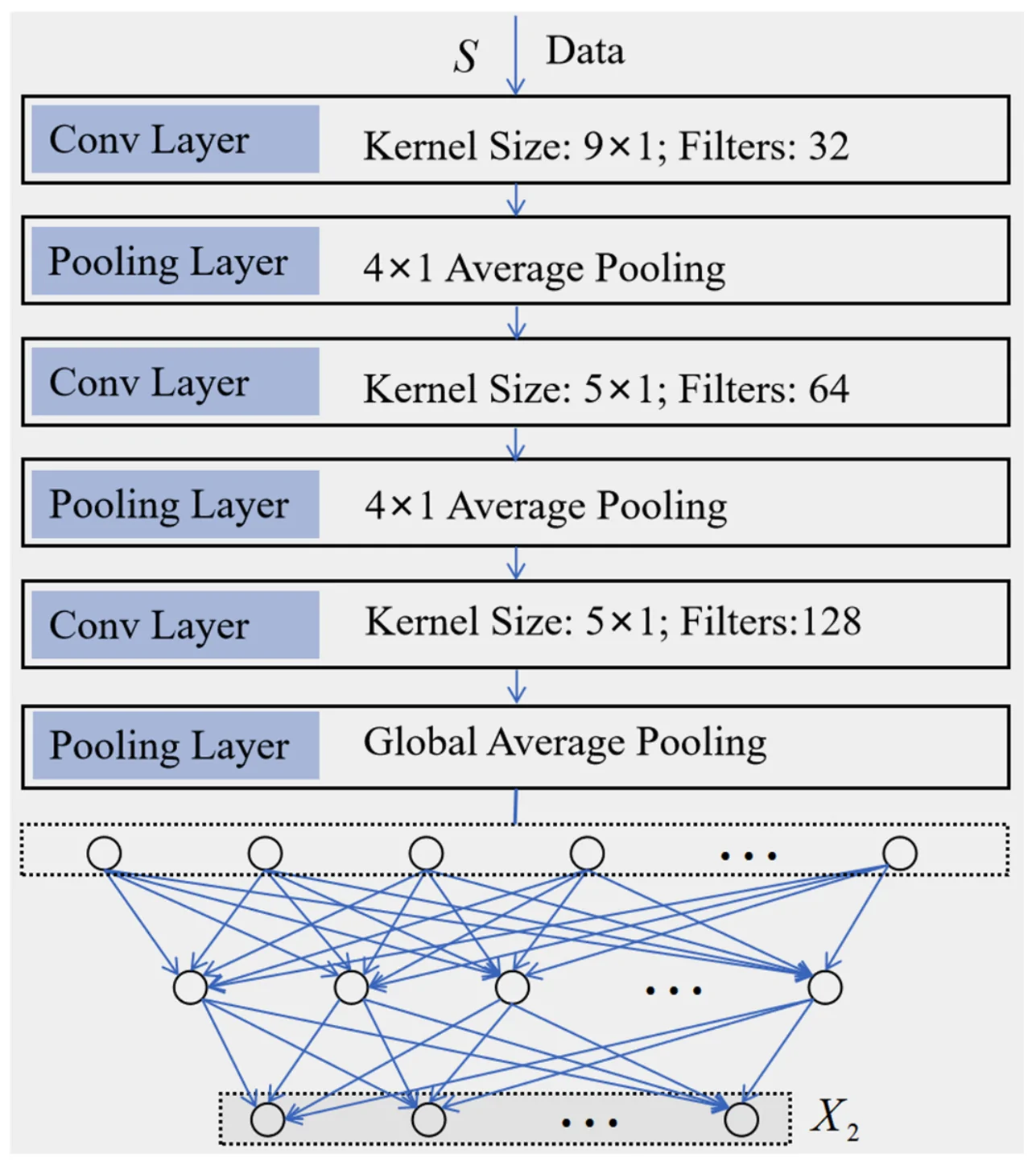
Architecture of 1DCNN.

The 1D convolutional neural network (1DCNN) shown in Figure 5 comprises 3 convolutional layers and 3 pooling layers. The kernel size, number of channels, and type of pooling operation for each layer are presented in the figure. Following these layers, three fully connected layers are cascaded, with the numbers of neurons set to 128, 64, and 20 respectively; these serve to compress the data into 20-dimensional features.

**Figure 6:**
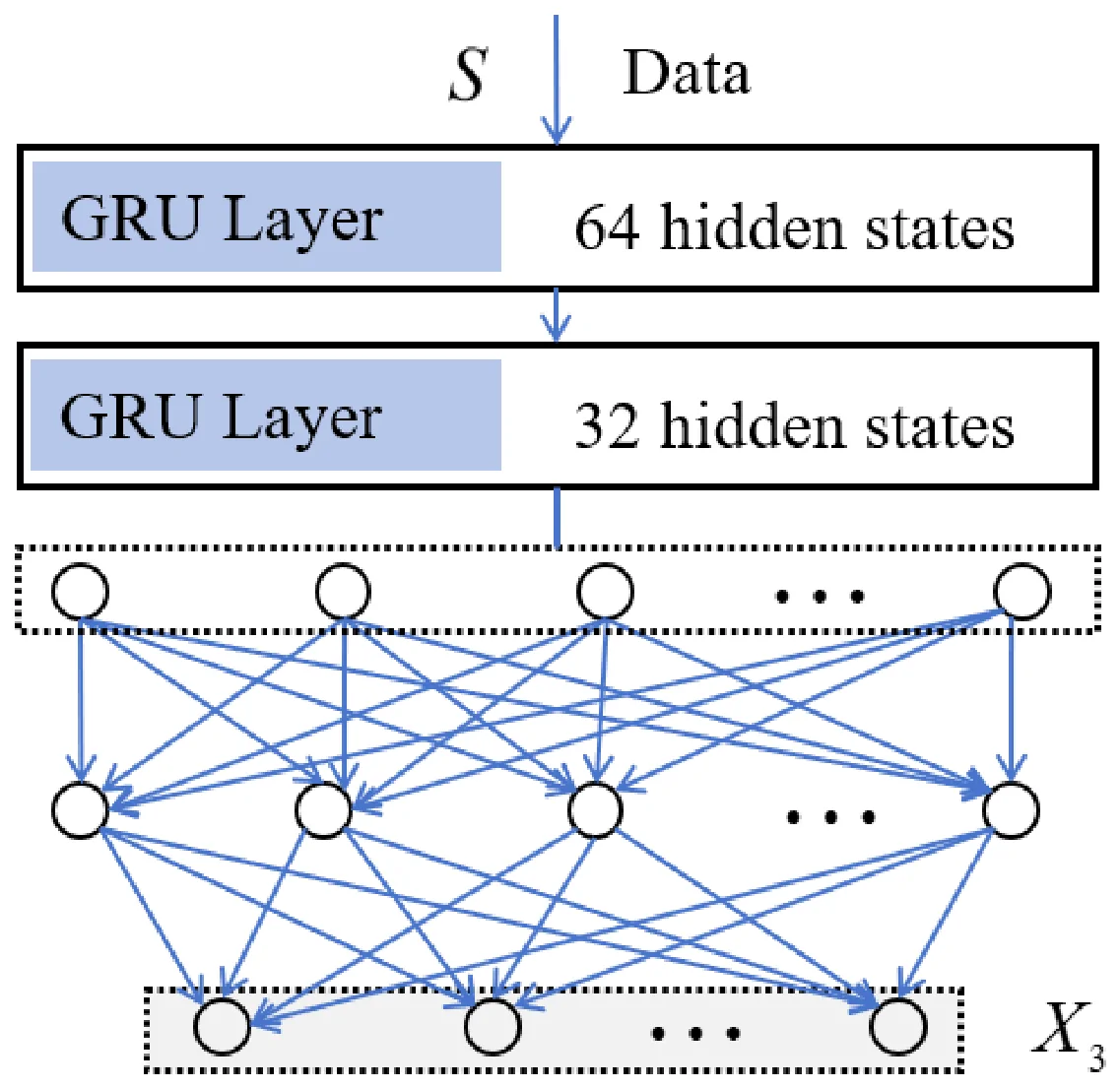
Architecture of GRU.

The Gated Recurrent Unit (GRU) shown in Figure 6 includes 2 GRU layers, where the number of hidden states for each layer is provided in the figure. Subsequent to the GRU layers, three fully connected layers are connected, with 32, 32, and 20 neurons respectively, used to compress the data into 20-dimensional features.

### 3.3. Feature Fusion Module

The fusion module first concatenates the three features extracted separately in the above subsection. These concatenated features are then fed in parallel into a linear transformation and a bilinear transformation. The linear transformation accounts for linear combination of features, which is widely used in feature fusion. The bilinear transformation considers correlation among features, and it can model pairwise feature interactions, which is particularly useful for fine-grained categorization [19]. These two modules explicitly model these two distinct relationships. The resulting representations are then concatenated and passed through the compression unit, which integrates all the aforementioned relationships, further extracts deeper features, and reduces the feature dimension to 30.

The feature compression is performed under the Information Bottleneck (IB) criterion [16,17,18], which is formulated as the following optimization problem:

$${\min L}_{\mathrm{IB}}\;=\min\left[I\left(X;Z\right)-\beta I\left(Z;Y\right)\right].$$

This objective minimizes the mutual information *I* between the input *X* and the feature *Z*, thereby compressing redundant information as much as possible. At the same time, it maximizes the mutual information *I* between *Z* and the label *Y*, thus preserving as much information as possible that is relevant to determining the sample category. The former compresses the input to the classification network, simplifying the classification task, while the latter retains information pertinent to sample classification. Both are beneficial for improving classification accuracy.

### 3.4. Classifier

The classifier is simply a Multi-Layer Perceptron (MLP) with a single hidden layer, where the numbers of neurons in the three layers are 30, 128, and 5, respectively. The five neurons in the output layer employ the softmax activation function, with each neuron corresponding to the predicted probability $p_{i,c}$ of a specific province. The sum of the output values from these five neurons is constrained to 1. During the inference phase, the province corresponding to the maximum output probability is determined as the predicted origin of the sample.

### 3.5. Network Training Method

First, the three feature extraction modules are pre-trained independently. The autoencoder is pre-trained via input reconstruction, while the 1DCNN and GRU networks are pre-trained via sequence reconstruction. 

Cross-entropy loss is adopted as the objective function for fine-tuning and is defined as

$$L_{\mathrm{CE}}\;=-\frac{1}{N}\sum_{i=1}^{N}\sum_{c=1}^{C}y_{i,c}\log\left(p_{i,c}\right).$$

This loss function is jointly optimized with the Information Bottleneck objective function, and the overall objective function can be formulated as

$$L={\;L}_{\mathrm{CE}}\;+\alpha L_{\mathrm{IB}},$$

where α is a hyperparameter set simply to 0.1 in the subsequent experiments.

Since the Information Bottleneck objective function is not amenable to direct optimization, we adopt the variational approximation method proposed in [18]. Following [18], we use $q_{\phi }\left(z|x\right)$ to denote the variational encoder, which maps x to the parameters of a diagonal Gaussian distribution:

$$q_{\phi }\left(z|x\right)=N\left(\mu_{\phi }\left(x\right),diag\left(\sigma_{\phi }^{2}\left(x\right)\right)\right).$$

The reference distribution for the encoded representation Z is chosen as a standard Gaussian distribution:

$$r\left(z\right)=N\left(0,I\right).$$

We further introduce $q_{\psi }\left(y|z\right)$ as a decoder. Then the mutual-information terms are bounded as follows [18]:

$$I\left(X;Z\right)\leq E_{X}\left[D_{KL}\left(q_{\phi }\left(z|x\right)∥r\left(z\right)\right)\right],$$

$$I(Z;Y)\geq H(Y)+E\left[log\;q_{\psi }\left(y|z\right)\right],$$

where $D_{KL}$ denotes the Kullback–Leibler divergence. Since $H\left(Y\right)$ is independent of the model parameters, it can be omitted during optimization, yielding the variational IB loss

$$L_{VIB}=E_{X}\left[D_{KL}\left(q_{\phi }\left(z|x\right)∥r\left(z\right)\right)\right]-\beta E\left[log\;q_{\psi }\left(y|z\right)\right].$$

We call the first term and the second term the KL term and the prediction term respectively. Under the Gaussian assumptions above, the KL term admits the following closed-form expression, which provides a tractable objective for optimizing the variational encoder:

$$D_{KL}\left(q_{\phi }\left(z|x\right)∥r\left(z\right)\right)=\frac{1}{2}\sum_{j}\left[\mu_{\phi ,j}^{2}\left(x\right)+\sigma_{\phi ,j}^{2}\left(x\right)-log\;\sigma_{\phi ,j}^{2}\left(x\right)-1\right].$$

For the prediction term, with one-hot labels, it can be estimated in the same cross-entropy form as $L_{CE}$.

Though the prediction term is functionally equivalent to cross-entropy loss, we still retain the separated cross-entropy loss $L_{CE}$ in the overall objective function. This enables independent ablation of the IB without affecting the supervised learning. By using the reparameterization trick to bridge the variational encoder (the feature fusion module) and the decoder (the classifier), we can then optimize the objective function $L\;={\;L}_{\mathrm{CE}}\;+\;\alpha L_{\mathrm{VIB}}$ using the Stochastic Gradient Descent (SGD) algorithm, which is a well-established and commonly used method; thus, a detailed description is not elaborated here.

## 4. Experimental Results

### 4.1. Qualitative Results

The dataset was partitioned randomly into a training set and a testing set in proportions of 80% and 20%, respectively. The model was trained in a mini-batch fashion, where each batch contained 64 randomly selected samples. This is a classical mini-batch size that achieves a favorable balance between gradient stability and training stochasticity during the training process.

**Figure 7:**
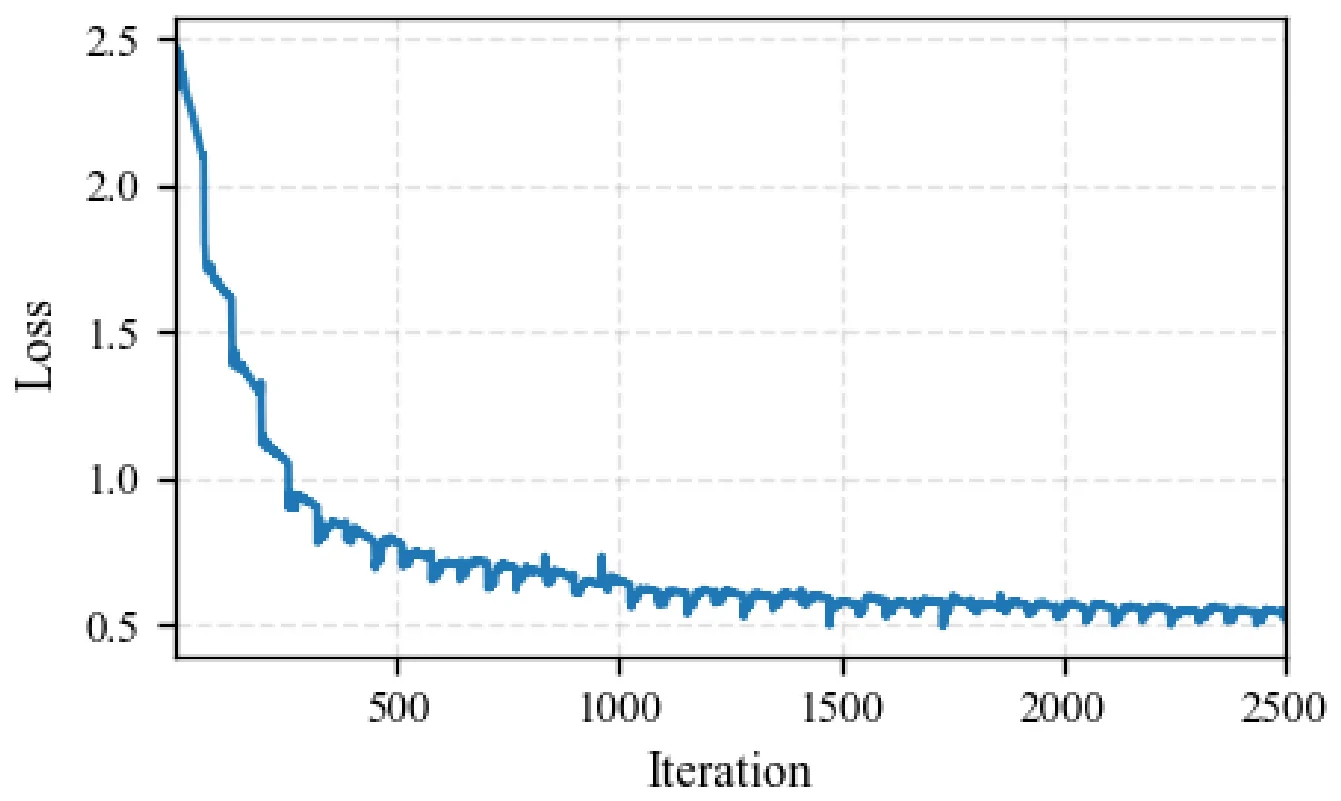
The training loss curve of the model.

We plotted the training curve with the iteration number as the horizontal axis and the objective function *L* as the vertical axis, as shown in Figure 7. It can be observed that the loss decreased gradually with an increase in iterations, and the learning curve converged after approximately 1500 iterations.

**Figure 8:**
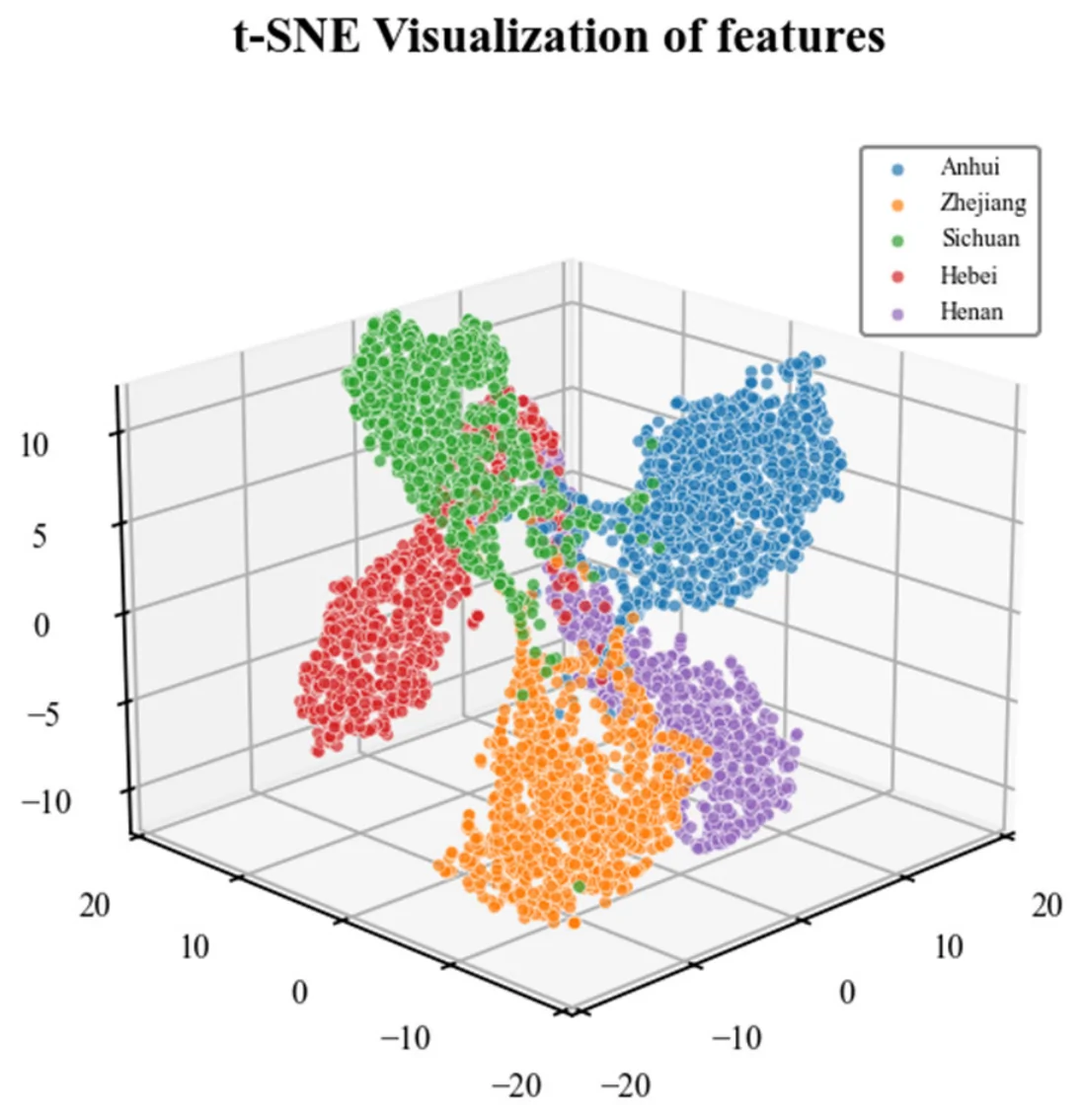
3D t-SNE visualization of the fused hyperspectral features.

After convergence, we performed t-SNE visualization on the fused 30-dimensional features of all samples, the results of which are presented in Figure 8. A significant difference can be observed when these results are compared with the t-SNE visualization results for the original hyperspectral data in Figure 1. The extracted and fused features exhibit favorable separability: samples from each province are basically clustered together and occupy distinct spatial positions from those of other provinces. This verifies the effectiveness of the feature extraction and fusion method proposed in this paper, which also facilitates the subsequent classification task. 

The Silhouette score, used as a quantitative metric, was also computed for the fused features and the raw data, yielding values of 0.7431 and 0.0098, respectively. This further confirms the above conclusion.

### 4.2. Quantitative Experiments

In this section, we describe quantitative experiments where the performance was evaluated using the classification accuracy on the test data. For each experimental setting, we repeated the experiment 20 times, with the training and test data randomly partitioned in each run. The average classification accuracy is reported as the final accuracy in the tables.

#### 4.2.1. Ablation Study

In this set of experiments, we selected 80% of the data as the training set and the remaining 20% as the test set. We evaluated the performance of using each feature extractor individually, pairwise combinations of feature extractors with IB-based fusion, and all three feature extractors with IB-based fusion. The extracted features were then fed into the classifier, and the results are listed in the first seven rows of Table 1. It can be clearly seen from the table that the accuracy achieved by using all three feature extractors with fusion was the highest, reaching 96.83%. The accuracy of pairwise fusion was lower than that of three-way fusion, and the performance of individual feature extractors dropped further. This indicates that the three feature extractors exhibit certain complementarity and are all indispensable.

In the last row of the table, we present the results of directly concatenating the features extracted by the three feature extractors without IB-based fusion. It can be observed that its accuracy was lower than that with IB fusion. This may be attributed to the high dimensionality of the concatenated features, which makes the classification task more difficult for the classifier. Moreover, without the constraint of IB, the complementarity of the information extracted by the three feature extractors is not guaranteed. This demonstrates the necessity of the Information Bottleneck.

#### 4.2.2. Training Under Limited Data

In this part, we changed the experimental conditions by selecting 20% of the data as the training set and another 20% as the test set to investigate the learning performance under limited training samples. The results are listed in Table 2.

From the table, we observe that when the training data is limited, the performance of individual feature extractors drops significantly, and the performance of the multi-view method also decreases slightly but still achieves relatively satisfactory results. This result can be interpreted as follows: When the training data is insufficient, individual feature extractors cannot be fully trained, leading to suboptimal extracted features. However, the feature fusion mechanism can fuse several suboptimal features into a more discriminative one, thus maintaining relatively good performance. The performance in the last row drops more significantly, which may be due to the high dimensionality of the concatenated features from the three extractors, which, combined with the limited training data, makes it more difficult to train a well-performing classifier.

## 5. Conclusions

Accurate classification of the geographical origins of Chinese medicinal herbs is a core step in ensuring their quality and clinical efficacy, and hyperspectral imaging technology offers an efficient and non-destructive technical approach for this task. This paper proposed a multi-perspective hyperspectral classification method for *Angelica dahurica* based on Information Bottleneck (IB) feature fusion. This method treats hyperspectral data as both tabular and spectral sequence data and extracts complementary features using an autoencoder (AE), a 1D convolutional neural network (1DCNN), and a Gated Recurrent Unit (GRU). The extracted multi-perspective features are further fused by a fusion network following the IB criterion and then input into a three-layer Multi-Layer Perceptron (MLP) for origin classification.

Qualitative and quantitative experimental results on hyperspectral data from *Angelica dahurica* validated the effectiveness of the proposed method: the training curve converged stably after approximately 1500 iterations, and t-SNE visualization of the fused features demonstrated significant inter-class separability. Ablation experiments showed that the three-view feature fusion scheme yielded the highest classification accuracy, significantly higher than that of the single-view and pairwise fusion schemes, thus verifying the complementarity of the three feature extractors. A comparison with the direct feature concatenation scheme further illustrated the necessity of IB-based fusion for reducing feature dimensionality and ensuring information complementarity. Even with limited training data (20% for the training set), the proposed method still achieved relatively satisfactory accuracy, far higher than that of the direct concatenation scheme, reflecting its strong generalization ability in small-sample scenarios.

In summary, the multi-view feature fusion method based on the IB criterion can fully exploit the rich information contained in hyperspectral data and effectively improve the accuracy of geographical origin classification for Chinese medicinal herbs, thereby providing a new technical reference for the quality identification and origin classification of such herbs. The proposed framework can be applied to hyperspectral classification tasks for other species of Chinese medicinal herb.

## Figures and Tables

**Table 1:** Classification accuracy under different feature extraction and fusion strategies.

Feature Extractor With/Without IB	Accuracy
AE	0.9383
1DCNN	0.9436
GRU	0.9392
AE + 1DCNN with IB	0.9594
AE + GRU with IB	0.9515
1DCNN + GRU with IB	0.9550
AE + 1DCNN + GRU with IB	0.9683
AE-1DCNN-GRU concatenation	0.9548

**Table 2:** Classification accuracy under different feature extraction and fusion strategies for training under limited data.

Feature Extractor With/Without IB	Accuracy
AE	0.8739
1DCNN	0.8757
GRU	0.8933
AE + 1DCNN with IB	0.9136
AE + GRU with IB	0.9223
1DCNN + GRU with IB	0.9233
AE + 1DCNN + GRU with IB	0.9418
AE-1DCNN-GRU concatenation	0.9127

## Data Availability

The raw data supporting the conclusions of this article will be made available by the authors on request.
